# RACK1 Specifically Regulates Translation through Its Binding to Ribosomes

**DOI:** 10.1128/MCB.00230-18

**Published:** 2018-11-13

**Authors:** Simone Gallo, Sara Ricciardi, Nicola Manfrini, Elisa Pesce, Stefania Oliveto, Piera Calamita, Marilena Mancino, Elisa Maffioli, Monica Moro, Mariacristina Crosti, Valeria Berno, Mauro Bombaci, Gabriella Tedeschi, Stefano Biffo

**Affiliations:** a Molecular Histology and Cell Growth Unit, National Institute of Molecular Genetics Romeo e Enrica Invernizzi (INGM), Milan, Italy; b Università Vita-Salute San Raffaele, Milan, Italy; c Translational Research Unit, National Institute of Molecular Genetics Romeo e Enrica Invernizzi (INGM), Milan, Italy; d Filarete Foundation, Milan, Italy; e DIMEVET, University of Milan, Milan, Italy; f Flow Cytometry Facility, National Institute of Molecular Genetics Romeo e Enrica Invernizzi (INGM), Milan, Italy; g Imaging Facility, National Institute of Molecular Genetics Romeo e Enrica Invernizzi (INGM), Milan, Italy; h Protein Array Unit, National Institute of Molecular Genetics Romeo e Enrica Invernizzi (INGM), Milan, Italy; i DBS, University of Milan, Milan, Italy

**Keywords:** PKC, RACK1, cell signaling, eIF4E, eIF6, mRNA, puromycin, ribosomes, stress, translational control

## Abstract

The translational capability of ribosomes deprived of specific nonfundamental ribosomal proteins may be altered. Physiological mechanisms are scanty, and it is unclear whether free ribosomal proteins can cross talk with the signaling machinery. RACK1 (receptor for activated C kinase 1) is a highly conserved scaffold protein, located on the 40S subunit near the mRNA exit channel. RACK1 is involved in a variety of intracellular contexts, both on and off the ribosomes, acting as a receptor for proteins in signaling, such as the protein kinase C (PKC) family. Here we show that the binding of RACK1 to ribosomes is essential for full translation of capped mRNAs and efficient recruitment of eukaryotic initiation factor 4E (eIF4E). *In vitro*, when RACK1 is partially depleted, supplementing the ribosome machinery with wild-type RACK1 restores the translational capability, whereas the addition of a RACK1 mutant that is unable to bind ribosomes does not. Outside the ribosome, RACK1 has a reduced half-life. By accumulating in living cells, free RACK1 exerts an inhibitory phenotype, impairing cell cycle progression and repressing global translation. Here we present RACK1 binding to ribosomes as a crucial way to regulate translation, possibly through interaction with known partners on or off the ribosome that are involved in signaling.

## Abstract (precis)

The translational capability of ribosomes deprived of specific nonfundamental ribosomal proteins may be altered. Physiological mechanisms are scanty, and it is unclear whether free ribosomal proteins can cross talk with the signaling machinery.

## INTRODUCTION

Ribosomes are rate-limiting for translation (1). It has long been held that an insufficiency of ribosomal proteins leads to a reduction in the general rate of translation without affecting any specific mRNA. The structural resolution of eukaryotic ribosomes has provided an unprecedented view of their complexity. In particular, specific ribosomal proteins are located close to precise mRNA sequences or interact with eukaryotic initiation factors (eIFs) (2). This observation supports the hypothesis that some ribosomal proteins can affect the translation of specific mRNAs (3, 4). In addition, ribosomes offer large rRNA surfaces, which have expanded upon evolution (5), suggesting that some ribosomal surfaces may also act as local signaling hubs. Alternatively, a quantitative model predicts that a reduction in the number of ribosomes may specifically affect the translation of inefficient mRNAs through increased ribosomal competition (6).

A ribosomal protein that may control both mRNA-specific translation and signaling is RACK1 (receptor for activated C kinase 1). RACK1 binds 40S ribosomal subunits close to the mRNA exit channel (7). Genetic depletion of RACK1 orthologs in yeast cells is not lethal (8), but it leads to a puzzling lack of adaptive responses during amino acid deprivation, which include reduced survival and defective Gcn2-mediated eIF2α phosphorylation (9). Further *in vivo* studies on Saccharomyces cerevisiae yeast show that the translation of small open reading frames (ORFs) is sensitive to depletion of the RACK1 homolog Asc1 (10), whereas in Drosophila melanogaster, RACK1 depletion affects internal ribosomal entry site (IRES)-mediated translation (11). These data suggest that RACK1 regulates both the translation of specific mRNAs and the interface between translational and regulatory networks, rather than being essential for translation itself. In mammals, depletion of RACK1 is embryonically lethal at the late-gastrulation stage (12), supporting the notion that RACK1 is not strictly necessary for translation but demonstrating that it has acquired critical regulatory functions or that it regulates the translation of specific mRNAs. We lack *in vitro* models in which the direct function of RACK1 on ribosomes is tested. Measurement of the translational efficiency of mammalian ribosomes depleted of RACK1, *in vitro*, with controlled readministration of RACK1 may define the specific activity of RACK1-depleted ribosomes, without the bias of indirect effects.

RACK1 was originally isolated as a receptor for activated protein kinase C (PKC) (13). In this context, RACK1 may act as a signaling complex with PKC to activate the translational activity of eIF6 (14–16). RACK1 inactivation partly phenocopies eIF6 depletion with respect to impaired stimulation of protein synthesis after PKC activation (12, 17). However, gene expression studies have shown that the expression signature elicited by RACK1 depletion is different from that induced by eIF6 depletion (18), indicating that RACK1 and eIF6 also have independent functions. RACK1 may be essential for the degradation of newly synthesized polypeptides (19), in accord with a role in coordinating translation-related events.

In general, many reports have described extraribosomal functions of RACK1 that modulate different pathways in different cells (20, 21). Unfortunately, we lack a unifying model of the role of RACK1 in regulating specific translation and the mechanism by which its extraribosomal activities connect to its signaling properties and translation.

We addressed the role of RACK1 in specific translation and its connection to signaling. We first developed an *in vitro* system in which we showed that the binding of RACK1 to ribosomes is necessary for cap-dependent translation. We then found that when not bound to the ribosome, RACK1 is unstable and still impacts the cellular phenotype by inhibiting cell cycle progression and translation. Here we present RACK1 as a multifaceted protein that is able to shape phenotypes in different ways, particularly with respect to translation, depending on its ribosome-binding status.

## RESULTS

### Efficient translation of capped mRNAs requires RACK1.

**FIG 1:**
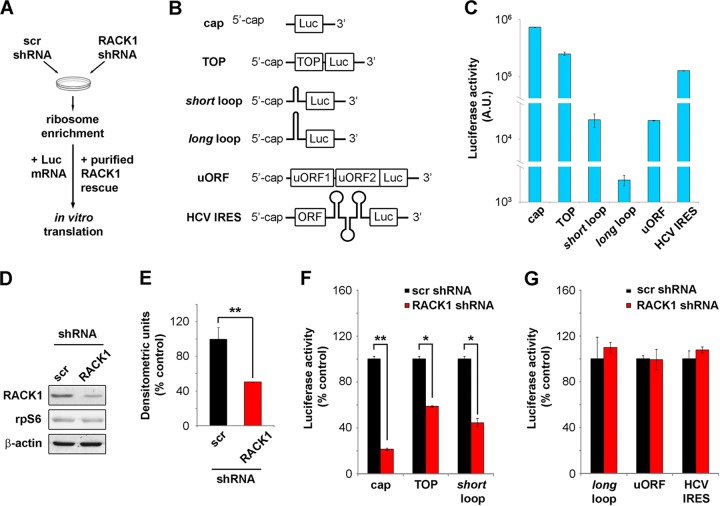
RACK1 is essential for efficient translation of capped mRNAs *in vitro*. (A) Scheme representing the *in vitro* translation strategy used. (B) Diagrams of the mRNA reporters employed. (C) Absolute luciferase counts from *in vitro* translation of the reporters. Values are shown on a logarithmic scale. A.U., arbitrary units. (D) Representative Western blot assessing RACK1 protein depletion in samples used for *in vitro* translation. scr, scrambled sequence. (E) Quantification of RACK1 protein in the samples. RACK1 protein levels were normalized to β-actin levels. (F) Quantification of the translational efficiency, *in vitro*, of cap-, TOP-, and *short* loop-regulated mRNA reporters under conditions of RACK1 downregulation. (G) Quantification of the *in vitro* translational outputs of *long* loop-, uORF-, and HCV IRES-regulated reporters upon RACK1 downregulation. Data are from a representative assay. At least four independent replicates were performed for each assay. Means and standard deviations are shown. Statistical significance was determined by the *t* test. *P* values are indicated as follows: *, <0.05; **, <0.01.

RACK1 is a scaffold protein whose interactome includes multiple partners involved in many cellular processes (22), e.g., signal transduction (13), translation (21), adhesion (23), and quality control for mRNA translation (24) and nascent polypeptides (19). The most stable and consistent interaction of RACK1 is that with the ribosome. Indeed, RACK1 is found on 40S ribosomal subunits (14) next to the mRNA exit channel (25). Possibly owing to its position on the ribosome and to its interaction capabilities, RACK1 specifically modulates translational efficiency in various models (10–12, 26). However, we still lacked a systematic characterization of the mRNA classes that depend on RACK1 for efficient translation. In order to address this fundamental point, we adapted an assay based on a cell-free system (27) that recapitulates the translation process *in vitro*. The approach is based on preparing ribosomes partly depleted of RACK1, followed by *in vitro* reconstitution with physiological amounts of RACK1 (Fig. 1A).

We prepared luciferase-encoding mRNA reporters with specific regulatory features in *cis* (Fig. 1B) and compared their translational efficiencies in HeLa cell extracts. We analyzed different 5′ regions, including a nonstructured capped 5′ mRNA (5′-GGCTAGCCACCATG-3′), an mRNA with a 5′-terminal oligopyrimidine tract (TOP) (28), two stem-loops of different unfolding energies (see Materials and Methods), an upstream open reading frame (uORF) sequence derived from the 5′ untranslated region (5′ UTR) of ATF4 mRNA (29), and the HCV IRES (30). We performed *in vitro* translation reactions with identical amounts of mRNA and monitored translational efficiency by measuring luciferase activity. The absolute luciferase counts show that the translation of equal amounts of mRNAs results in diverse protein outputs, clearly depending on their 5′ sequences (Fig. 1C), thus validating our model. Specifically, the cap-presenting reporter was most efficiently translated, followed by the TOP mRNA (∼3-fold less efficient), the HCV IRES-containing mRNA (∼6-fold less efficient), and the shorter-loop-containing reporter and uORF-containing mRNA (both ∼33-fold less efficient).

The cell-free system was then used to directly assess the role of RACK1 in translation by preparing ribosomal extracts from cells depleted of RACK1. We prepared HeLa S10 cells transduced with lentiviral vectors expressing either a combination of three RACK1 short hairpin RNAs (shRNAs) or a scrambled sequence, and we characterized the general changes in cellular viability. The extent of RACK1 protein downregulation, as estimated by Western blotting, was around 50% (as shown by a representative blot in Fig. 1D and by quantification in Fig. 1E). Levels of the 40S ribosomal protein rpS6 were unchanged, in line with the fact that RACK1 depletion does not affect 40S ribosomal biogenesis.

After preparing ribosomal extracts from RACK1-depleted cells, we performed *in vitro* translation assays with fixed amounts of reporter mRNAs. We found that upon RACK1 depletion, cap-, TOP-, and *short* loop-regulated mRNAs were translated with ∼6-, ∼1.5-, and ∼2.5-fold less efficiency, respectively, than the control (Fig. 1F). Under the same conditions, the translation of HCV IRES- and uORF-containing mRNAs was not impaired (Fig. 1G).

### RACK1 binding to ribosomes is required for efficient translation and optimal eIF4E recruitment.

**FIG 2:**
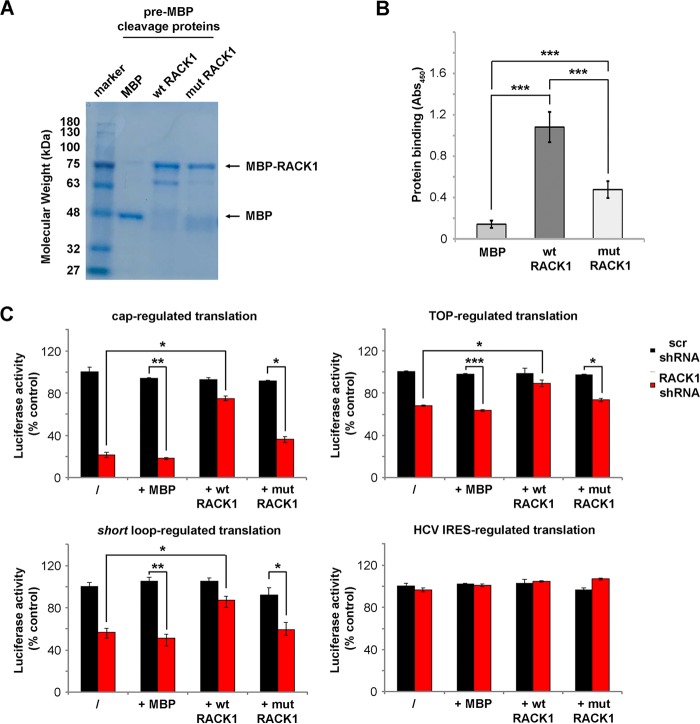
RACK1 binding to the ribosome is necessary for modulating translation. (A) Representative Coomassie blue-stained gel showing purified MBP-RACK1 proteins prior to MBP cleavage. wt, wild type; mut, mutant. (B) Efficiencies of binding of recombinant proteins (MBP [used as a control], wild-type RACK1, and R36D K38E mutant RACK1) to the ribosome as measured by an *in vitro* ribosome interaction assay. (C) Quantification of *in vitro* translational efficiencies of different mRNAs under conditions of RACK1 depletion that were rescued by recombinant wild-type or mutant RACK1 proteins. Data are from representative assays. At least four independent replicates per experiment were performed. Means and standard deviations are shown. Statistical significance was determined by the *t* test. *P* values are indicated as follows: *, <0.05; **, <0.01; ***, <0.001.

The translational deficit observed in extracts of RACK1-depleted cells could be due to indirect effects or to a direct effect on translation. In addition, it is unclear whether binding to ribosomes is required to sustain the effect of RACK1. We therefore asked if the role of RACK1 in translation depends on its binding to the ribosome or on a ribosome-independent function. We prepared wild-type RACK1 and the R36D K38E mutant (Fig. 2A), which binds the 40S ribosomal subunit at lower efficiency (31), as assessed by an *in vitro* ribosome interaction assay (*i*RIA) (32) (Fig. 2B). *In vitro* reaction mixtures were supplemented with 100 ng of either wild-type or mutant RACK1/assay (∼5-fold the amount of the endogenous protein) in order to saturate the ribosomes with recombinant RACK1. Exogenous wild-type RACK1 restored the impaired translation of RACK1-regulated mRNA reporters, while the R36D K38E mutant did not (Fig. 2C). The translational efficiencies of the mRNA reporters that were not affected by RACK1 depletion, such as the HCV IRES-containing mRNA, did not change upon the addition of exogenous RACK1 (Fig. 2C, bottom right). Taken together, our results demonstrate that RACK1 binding to the ribosome is essential for the efficient initiation of cap-dependent translation.

**FIG 3:**
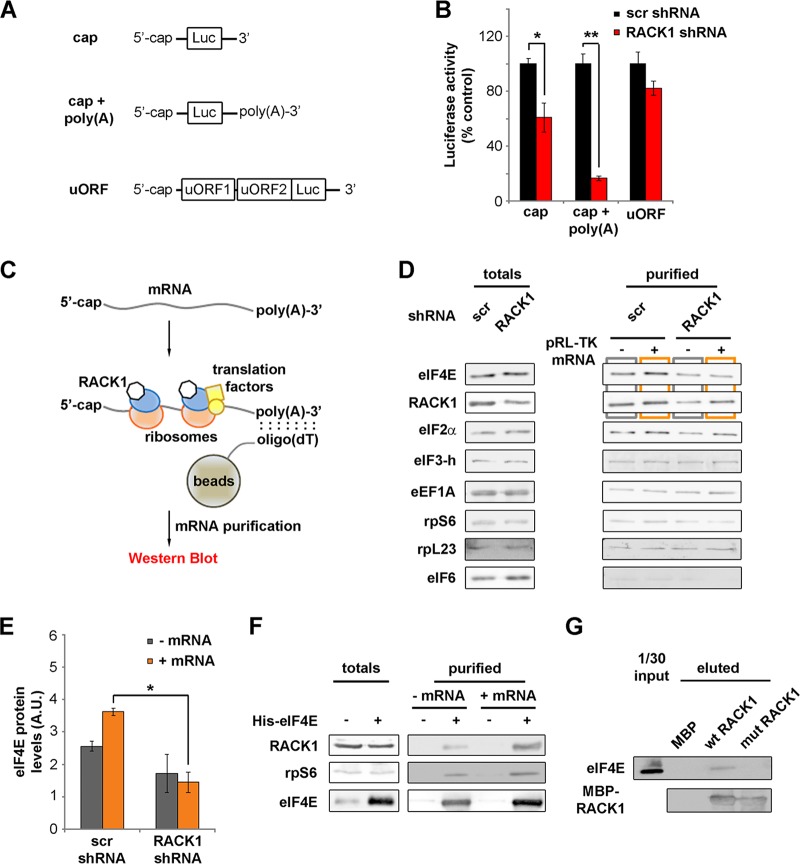
RACK1 on the ribosome recruits eIF4E. (A) Diagrams of mRNAs, with or without an *in vitro*-synthesized poly(A) tail, employed in the experiment for which results are shown in panel B. (B) *In vitro* translational efficiencies (expressed as percentages of the control value, taken as 100%) of RACK1-depleted mRNAs either with or without an *in vitro*-synthesized poly(A) tail. (C) Schematic representation of the purification strategy and analysis approach for mRNA-associated ribosomes and translation factors. (D) Western blots of translation factors and ribosomal proteins copurified with translating mRNAs *in vitro* under conditions of RACK1 depletion. (E) Quantification (from three independent replicates) of the levels of eIF4E copurifying with a cap-presenting pRL-TK mRNA as shown by Western blotting in panel D. (F) Western blotting for proteins from HeLa cell extracts copurifying with His-eIF4E *in vitro* under conditions of mRNA digestion (− mRNA) or mRNA reconstitution (+ mRNA). (G) Western blotting to assess the ability of eIF4E to interact with purified RACK1 *in vitro*. Representative Western blots are shown. In graphs, means and standard deviations are shown. Statistical significance was determined by the *t* test. *P* values are indicated as follows: *, <0.05; **, <0.01. At least four independent replicates per experiment were performed.

In eukaryotes, *in vivo* translation of capped mRNAs is increased by the presence of 3′ poly(A) tails. Therefore, we tested whether mRNA polyadenylation at the 3′ end can alter RACK1 effects on translation. To this end, the translational efficiencies of cap-presenting mRNAs with or without *in vitro*-synthesized 3′ poly(A) tails (Fig. 3A) were assessed as described above. The results show that RACK1 depletion impairs the translation of mRNA containing a cap and a 3′ poly(A) tail (Fig. 3B).

Among all our reporters, the translation of the cap-presenting mRNA was the most sensitive to RACK1 depletion. We therefore focused on the cap-regulated reporter with a 3′ poly(A) tail (as shown in Fig. 3A). We incubated the mRNA with *in vitro* translation-competent lysates and proceeded with mRNA purification using oligo(dT) beads. We then detected the presence of mRNA-associated proteins by Western blotting (Fig. 3C). The amounts of the factors tested in the total cell extract were independent of RACK1 levels, indicating that RACK1 did not affect their stability (Fig. 3D). Under conditions of RACK1 depletion, we observed a specific decrease in the amount of eIF4E copurifying with the mRNA, both in the presence and in the absence of exogenous mRNA (Fig. 3D; quantification in Fig. 3E). All the other proteins tested, such as ribosomal proteins (rpS6, rpL23), initiation factors (eIF2α and eIF3-h), and elongation factors (eEF1A), did not show differences due to RACK1 levels in oligo(dT) pulldown. Notably, eIF6, an interactor of RACK1 and a marker of the free 60S ribosomal subunit, was not detected upon oligo(dT) pulldown, in line with the fact that it does not associate with translation-competent ribosomes (33).

We tested whether eIF4E binds RACK1 and the 40S ribosome *in vitro*, either upon mRNA digestion or upon mRNA reconstitution and *in vitro* translation. Recombinant eIF4E added to total-HeLa-cell lysates copurifies with endogenous RACK1 and rpS6 (Fig. 3F). eIF4E-RACK1 interaction is more efficient in the presence of exogenous mRNA than in its absence, further suggesting that the interaction is mediated by the translation machinery. We then checked for the ability of RACK1 to bind eIF4E by *in vitro* pulldown. We incubated recombinant wild-type and R36D K38E mutant RACK1 with extracts of HEK293 cells. We found that without mRNA digestion, eIF4E copurifies more efficiently with wild-type than with mutant RACK1 (Fig. 3G). These data suggest that RACK1 recruits eIF4E via translating ribosomes.

In conclusion, we demonstrate that the binding of RACK1 to ribosomes is strictly required for efficient translation of capped mRNAs and for maximal eIF4E recruitment.

### Ribosome-free RACK1 has a limited half-life and inhibits cell cycle progression.

**FIG 4:**
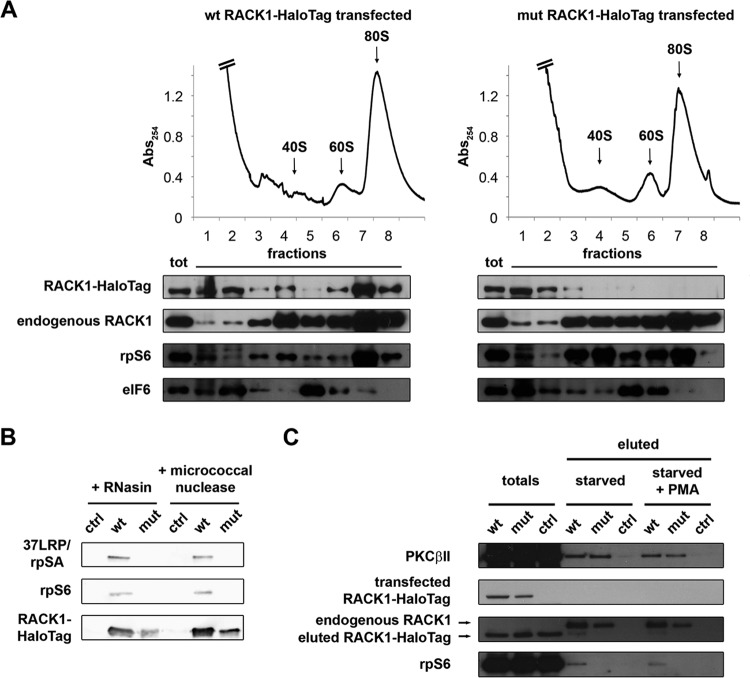
R36D K38E mutant RACK1 does not bind ribosomes efficiently. (A) Ribosome profiles coupled with Western blots show the distribution of RACK1-HaloTag in the profile fractions. rpS6 and eIF6 were used as markers of the 40S and 60S ribosomal subunits. Note the absence of mutant RACK1 from polysomes. (B) Western blotting was performed to assess the abilities of HaloTag-labeled wild-type and mutant RACK1 to copurify with 40S ribosomal proteins under conditions of mRNA degradation. (C) The ability of PKCβII to copurify with wild-type and mutant RACK1-HaloTag, independently of RACK1 binding to ribosomes, is shown by Western blotting on eluted samples. PMA, phorbol myristate acetate.

Having established that RACK1-deprived ribosomes in human cells are viable, we asked what might be the consequence of free RACK1 *in vivo*. Evidence from the literature (34) and our own data (Fig. 4A) shows that endogenous RACK1 can exist outside the ribosome. We thus hypothesized that free RACK1 has a physiological function(s) in cells. To evaluate the consequences of an accumulation of free RACK1 for the cellular phenotype, we expressed a HaloTag-labeled RACK1 R36D K38E mutant in HEK293 cells. In ribosome profile experiments, mutant RACK1 accumulated in the soluble fraction but did not alter the ability of endogenous RACK1 to bind to ribosomes (Fig. 4A). The level of exogenous RACK1 was always much lower than that of endogenous RACK1, ≅10% (Fig. 4A), thus excluding the possibility of artifacts due to overexpression. By protein purification followed by SDS-PAGE and Western blotting, we confirmed that mutant RACK1 does not interact efficiently with ribosomes (Fig. 4B). The well-known RACK1 interactor PKCβII (35) has been found to interact with the RACK1-40S ribosomal subunit complex *in vitro* (36). Further, the effect of RACK1 on translation is at least partially mediated by signaling, particularly with respect to typical PKCs (12, 15). We thus asked whether extraribosomal RACK1 retains its ability to bind PKC. By copurification experiments, we found that mutant RACK1 is able to interact with PKCβII (Fig. 4C), indicating for the first time that RACK1 can act as a PKC receptor independently of its binding to ribosomes.

**FIG 5:**
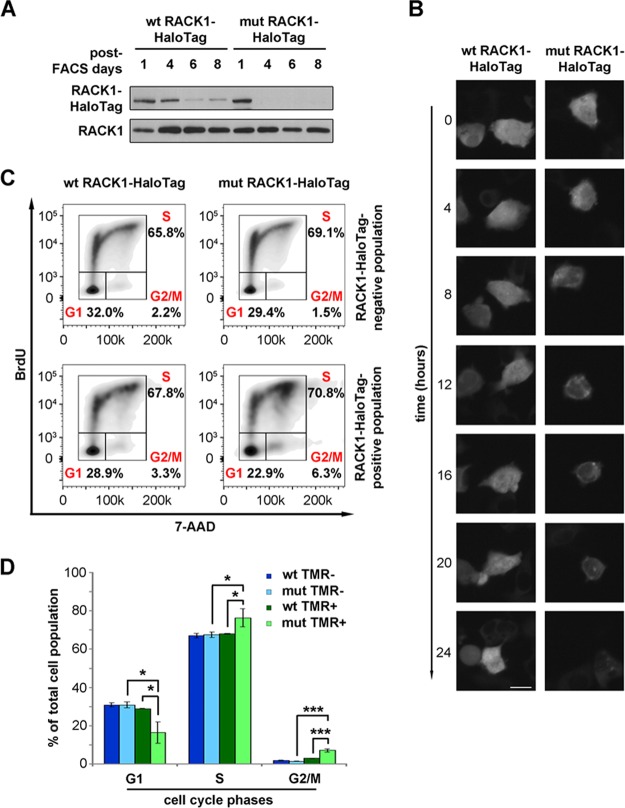
Unstable extraribosomal RACK1 inhibits cell cycle progression. (A) Western blotting to monitor the levels of wild-type and R36D K38E mutant RACK1 proteins in stably transfected cells after FACS purification of construct-positive populations. (B) Fluorescent marking of wild-type and mutant RACK1 constructs is shown at different time points in order to evaluate the half-lives of the proteins. Bar, 5 μm. (C) Representative FACS analysis of cells expressing wild-type or mutant RACK1 in the presence of BrdU incorporation. (D) Histogram showing the repartition of cell populations from panel C throughout the phases of the cell cycle. Data are means and standard deviations of results from three independent experiments. Statistical significance was determined by the *t* test. *P* values are indicated as follows: *, <0.05; ***, <0.001.

Importantly, we noticed that the levels of mutant RACK1 were lower than those of wild-type RACK1 (Fig. 4A and B), raising the question of whether free RACK1 has reduced stability. Most ribosomal proteins are unstable if not bound to ribosomes (37). We expressed wild-type or R36D K38E mutant RACK1 by stable transfection. After using fluorescence-activated cell sorting (FACS) to select stably transfected cells on the basis of construct expression, we monitored the amounts of RACK1 protein in cultured cells. The expression of mutant RACK1 was rapidly lost, in contrast to that of wild-type RACK1 (Fig. 5A), suggesting that either ribosomal binding stabilizes the protein or selective pressure occurs against cells expressing mutant RACK1. We then used live imaging to compare the half-lives of mutant and wild-type RACK1 under transient-transfection conditions. After 48 h from transfection, we labeled the cells *in vivo* with the HaloTag tetramethyl rhodamine (TMR) ligand and followed them by time-lapse fluorescence microscopy. Analyzing a 24-h time course, we found that fluorescence decreased faster in cells expressing mutant RACK1 than in cells expressing wild-type RACK1 (Fig. 5B).

Next, we performed a general characterization of cells expressing ribosome-free R36D K38E mutant RACK1 compared to cells expressing wild-type RACK1. Briefly, by FACS analysis for cell cycle progression, we found that mutant RACK1 expression causes a small but consistent accumulation of cells at the G_2_/M phase transition (Fig. 5C and D). We conclude that free RACK1 has a shorter half-life than ribosome-bound RACK1 and inhibits cell cycle progression.

### Accumulation of free RACK1 results in diminished translational output.

**FIG 6:**
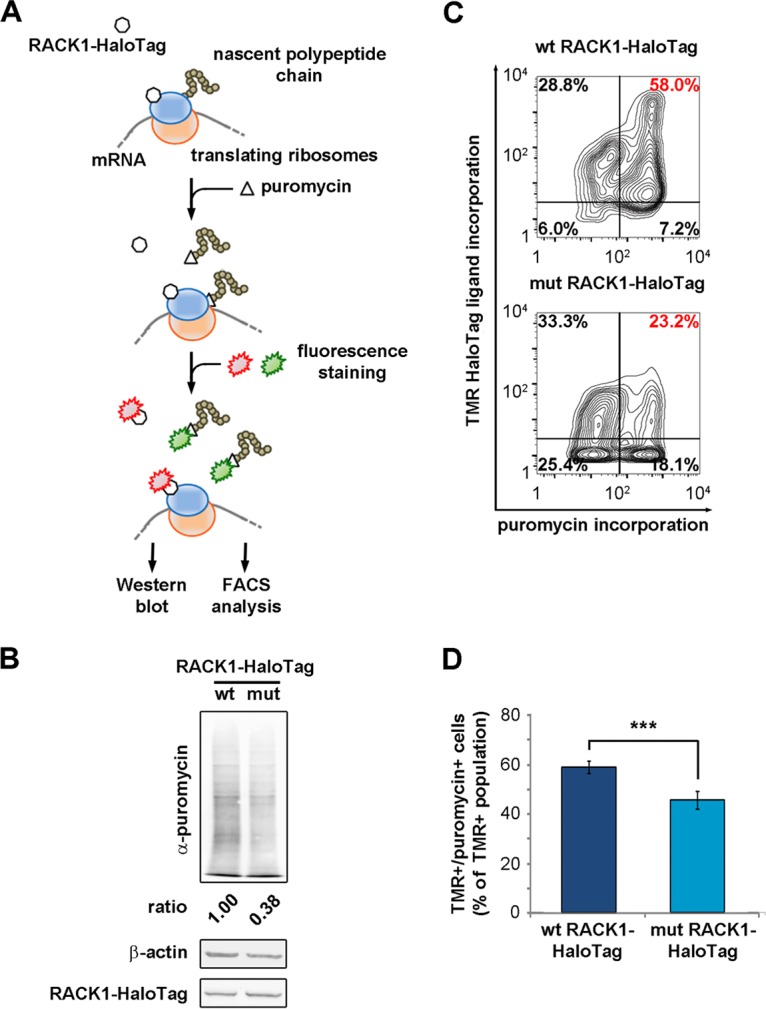
Free RACK1 represses translation in living cells. (A) Schematic representation of the puromycin incorporation assay used to quantify translation output at the single-cell level. (B) Quantitative Western blotting to detect the amounts of newly incorporated puromycin in lysates of RACK1-expressing cells. (C) Representative FACS analyses for puromycin incorporation correlate the translational rate and the expression of wild-type or mutant RACK1. (D) Comparison of the amounts of the TMR-positive, puromycin-positive populations from the experiment described in the text. Data are means and standard deviations of results from three independent experiments. Statistical significance (***, *P* < 0.001) was determined by the *t* test.

We then decided to test whether free RACK1 had any impact on translation in cells. We tested this by two approaches. After administering puromycin to cells expressing exogenous RACK1, we proceeded either (i) to lyse the cells and perform Western blotting or (ii) to mark incorporated puromycin with a fluorescent label different from the HaloTag and then perform FACS analysis (Fig. 6A). By the single-cell approach, we were able to avoid issues related to the measurement of translation rates in populations with various levels of RACK1 expression.

Our Western blots showed that the translational output was reduced in cells expressing mutant RACK1 (Fig. 6B). By FACS, we correlated translation levels to the expression of RACK1-HaloTag in single cells. Fluorescent labeling of RACK1-HaloTag and puromycin allowed us to distinguish between positive and negative populations for both. The double-positive population (HaloTag-positive, puromycin-positive RACK1) was reduced in samples transfected with mutant RACK1 (Fig. 6C and D), further confirming that extraribosomal RACK1 is able to repress translation. In short, free RACK1 can inhibit translation and cell cycle progression.

## DISCUSSION

A unifying model that reconciles ribosome-dependent and ribosome-independent functions of RACK1 has never been proposed. In this study, we addressed three questions: (i) do ribosomes devoid of RACK1 translate differentially? (ii) does RACK1 interact with specific translation factors on the ribosome? (iii) does RACK1 outside the ribosome “talk back” to the translational machinery? We show that ribosomes can translate capped mRNAs maximally only in the presence of RACK1, that eIF4E is recruited to ribosomes more efficiently in the presence of RACK1 than in its absence, and that RACK1 exists in a ribosome-free form with a limited half-life that represses translation. These data suggest that impairment of the binding of RACK1 to ribosomes, as under conditions of starvation (34), may repress translation through both ribosome-dependent and ribosome-independent mechanisms.

In this work, we have been able to prepare ribosomes partly devoid of RACK1 and to directly test the efficiency of such ribosomes under conditions of controlled RACK1 rescue. The data that we obtained show unequivocally that maximal translational capability depends on full levels of RACK1. RACK1 has also been found to interact with eIF4G (10). Recent data have shown that RACK1 can promote cap-dependent translation via eIF4G phosphorylation, also impacting eIF4E recruitment (38, 39).

Most of the results obtained so far by manipulating RACK1 expression (21) can now be explained by translational activation elicited by RACK1 through eIF6 (14) or eIF4E (this study), two rate-limiting eIFs downstream of growth factors (40), combined with PKC stabilization (13, 35) or the activation or inactivation of other signaling factors. For instance, our studies are in line with the effects of RACK1 depletion in knockout mice: postgastrulation lethality under homozygous conditions and blunted insulin-stimulated translation in heterozygosis (12). The fact that RACK1 depletion reduces the translation of capped mRNAs and the recruitment of eIF4E is in agreement with the fact that insulin-stimulated translation activates eIF4F formation (41) and induces eIF6 activity (17, 42).

With respect to a role for RACK1 in signaling, we confirmed the association of RACK1 with activated PKC (35) and demonstrated that the recruitment of PKC can occur independently of the binding of RACK1 to ribosomes. PKC-mediated phosphorylation is essential for the function of eIF6 (14, 16, 42). In the absence of phosphorylation, eIF6 may inhibit translation (14, 43). The availability of RACK1 for PKC interactions can thus be relevant, *in vivo*, in organs that contain high levels of both eIF6 and PKC (42, 44). However, alternative mechanisms for eIF6 activation are present during ribosome biogenesis, and these processes are not mutually exclusive (45).

Overall, it is noteworthy that the signal activated by free RACK1 is transient, because in the absence of ribosomal binding, the protein is unstable. In this respect, RACK1 behaves as a typical ribosomal protein that is stabilized by ribosomal binding and is translated as a TOP mRNA (46).

Deletion of *ASC1*, encoding the yeast homolog of RACK1, is not lethal at the single-cell level (10, 31), suggesting that the existence of a pool of RACK1-depleted ribosomes is compatible with life. The modulation of RACK1 expression or of its release from ribosomes therefore leads to ribosomal heterogeneity in cells. We demonstrated that RACK1-free ribosomes are translationally defective. We thus speculate that the ratio of RACK1-depleted ribosomes to wild-type ribosomes affects the translational output. As long as the ratio is low, wild-type ribosomes may compensate for the decreased translational efficiency of RACK1-depleted ribosomes. RACK1 function is therefore compatible with both quantitative and qualitative models that explain the effects of ribosomal protein depletion (6, 47).

**FIG 7:**
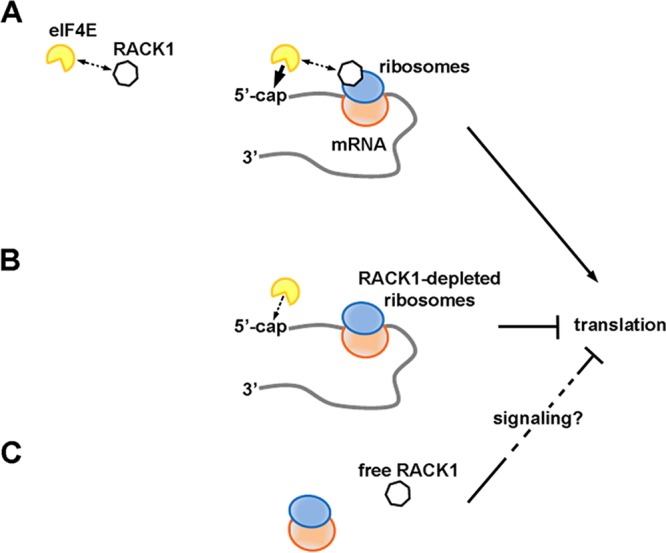
RACK1 has different effects on translation depending on its binding to the ribosome. (A) RACK1 can recruit eIF4E, possibly facilitating a well-timed association of eIF4E with the 5′ caps of mRNAs on translating ribosomes. This would increase translational levels. (B) RACK1-deprived ribosomes are less efficient at recruiting eIF4E, and their translational output is diminished. (C) Free RACK1, if released or not associated with the ribosome, can repress translation, possibly via signaling.

We propose eIF4E as a partner of RACK1 on translating ribosomes, possibly contributing to the RACK1-dependent upregulation of capped-mRNA translation. We speculate that even though RACK1 and eIF4E can interact, the complex they form is ultimately unstable. When RACK1-containing ribosomes translate, the capped 5′ end would be presented to RACK1-bound eIF4E near the mRNA exit channel. There, eIF4E could stably associate with the cap (Fig. 7A). This would result in the formation of an eIF4F complex efficiently coordinated at both the spatial and temporal levels, with ultimately increased translation. Conversely, ribosomes deprived of RACK1 would exhibit reduced eIF4E recruitment capability and translational output (Fig. 7B). Outside the ribosome, RACK1 can still impact translation, possibly indirectly, by activating inhibitory signaling pathways (Fig. 7C).

In conclusion, we propose that RACK1 is a crucial node in translation regulation and that its function depends on whether or not it binds to the ribosome. The association of RACK1 with the ribosome may indeed be regulated downstream of specific stimuli in order to modulate its functions in translation. Such a possibility is worth further investigation.

## MATERIALS AND METHODS

### Cell lines and microbial strains.

HEK293, HEK293T, and HeLa cells were cultured in Dulbecco's modified Eagle medium (DMEM; Lonza) supplemented with 10% fetal bovine serum (FBS) and 1% penicillin-streptomycin-glutamine (PSG) (both from EuroClone) at 37°C under 5% CO_2_.

Escherichia coli TOP10 and BL21(DL3) were cultured at 37°C in LB or were plated on LB agar and cultured at 37°C.

### Lentiviral vectors.

We used HEK293T to produce lentiviral vectors by transfecting the second-generation plasmids pMDG, CMV ΔR8.74, and pILLV (48) and either scrambled (sc-108060) or RACK1-targeting (sc-36354-SH) shRNA vectors (Santa Cruz Biotechnology). After 48 h from transfection, the conditioned medium was collected and either was used to incubate and infect cells or was stored at −80°C.

### Western blotting.

Equal amounts of proteins (20 μg per sample) from clarified lysates were loaded onto SDS-PAGE gels. After separation, the proteins were transferred by using the eBlot protein transfer system (GenScript) on polyvinylidene difluoride (PVDF) membranes. The membranes were blocked in 5% bovine serum albumin (BSA; Calbiochem) in phosphate-buffered saline (PBS) with 0.1% Tween 20 for 1 h before overnight incubation with primary antibodies. After washes in PBS–0.1% Tween 20, the membranes were incubated with appropriate horseradish peroxidase (HRP)-conjugated secondary antibodies (Santa Cruz Biotechnology). The signal was then developed with the Amersham ECL Prime Western blotting detection reagent (GE Healthcare), and the images were acquired with a LAS-3000 luminescent image analyzer (Fujifilm).

### Subcloning of luciferase-encoding plasmids.

Plasmid pRL-TK was obtained from Promega, and the whole sequence of the plasmid is available at http://www.ncbi.nlm.nih.gov/nuccore/7024220. We utilized pRL-TK in subsequent subclonings to obtain luciferase-encoding plasmids with sequences known to regulate the translation of specific mRNAs. The sequences chosen were as follows: (i) a TOP construct, featuring the TOP sequence present in the 5′ UTR of rpS6 (NCBI Reference Sequence Database accession no. NM_001010.2) (28); (ii) a *short* loop construct, featuring a stem-loop with a relatively high Δ*G* (−15 kcal/mol); (iii) a *long* loop construct, with a stem-loop with a lower Δ*G* (−41 kcal/mol) (29); (iv) a uORF construct, based on the ATF4 uORF (29).

With the exception of the *long* loop construct, sequences to be cloned were synthesized by the GeneArt Gene Synthesis service (Life Technologies). We then amplified the fragments by PCR, using PCR SuperMix, High Fidelity (Invitrogen), according to the manufacturer's instructions. The forward primers used in those PCRs were specific for each construct, while the reverse primers were shared (with the exception of the uORF construct).

The conditions of the reactions were as follows: for TOP PCR, 5 min at 95°C, 35 cycles composed of 30 s at 95°C, 30 s at 55°C, and 29 s at 72°C, and a final extension of 30 min at 72°C; for *short* loop PCR, 5 min at 95°C, 35 cycles composed of 30 s at 95°C and 59 s at 72°C, and a final extension of 30 min at 72°C; and for uORF PCR, 5 min at 95°C, 35 cycles composed of 30 s at 95°C, 30 s at 58°C, and 70 s at 72°C, and a final extension of 30 min at 72°C.

To obtain the *long* loop construct, we used a different strategy consisting of two overlap extension PCRs. The first PCR was performed with the first *long* loop forward primer and a *long* loop reverse primer. The PCR was designed with 5 min at 95°C, 35 cycles of 30 s at 95°C and 3 min at 72°C, and a final extension lasting 30 min at 72°C. We then performed the second PCR on the DNA amplified by the previous reaction using the second *long* loop forward primer and the *long* loop reverse primer from the previous reaction. The conditions of the reaction were the same as those in the previous step.

We then cloned all the DNA fragments obtained by PCR into an intermediate plasmid (pCR2.1-TOPO vector) using a TOPO TA cloning kit (Invitrogen) according to the manufacturer's instructions. We excised the DNA fragments of interest from the intermediate plasmid by restriction enzyme digestion. In particular, we used NheI to cut at the 5′ end and PciI at the 3′ end for the TOP, *short* loop, and uORF constructs, and we used NheI to cut at the 5′ end and BamHI at the 3′ end for the *long* loop construct. We digested the pRL-TK plasmid in the same way. We separated the digested samples by electrophoresis in Tris-acetate-EDTA (TAE)–agarose gels and purified the DNA of interest by employing the Wizard SV gel and PCR clean-up kit (Promega). We proceeded to ligate the purified fragments containing the sequences introduced by PCR and the backbone of the pRL-TK plasmid by using the Quick Ligation kit (New England BioLabs) according to the manufacturer's instructions.

### mRNA production and poly(A) tail synthesis *in vitro*.

We used linearized plasmids to obtain the corresponding mRNAs by using the MEGAscript T7 kit (Ambion) supplemented with the cap analog m^7^G(5′)ppp(5′)G (Ambion) at 2 mM to perform the *in vitro* transcription reaction. We used a poly(A) tailing kit (Applied Biosystems) to add poly(A) tails at the 3′ ends of the mRNAs.

### Purification of *in vitro*-translating mRNAs.

We incubated cap-presenting pRL-TK mRNA with *in vitro*-synthesized poly(A) tails on HeLa S10 cell extracts while reproducing the *in vitro* translation conditions of the other experiments. After 5 min from the start of the reaction, we added 0.36 mM cycloheximide and let the samples rest for 5 min. Then we loaded the samples onto Dynabeads Oligo(dT)_25_ (Invitrogen) that had been equilibrated in 100 mM Tris-HCl (pH 7.5), 100 mM LiCl, 0.66 mM EDTA, 0.66 mM Mg acetate, and 5 mM dithiothreitol (DTT) and incubated them for 5 min on a rotating wheel at room temperature. After removing the supernatant, we washed the beads on ice with equilibration buffer. Then we eluted the proteins that had copurified with the mRNA with Laemmli buffer. We separated the proteins by SDS-PAGE and performed Western blotting.

### *In vitro* translation assay.

We adapted a protocol using HeLa S10 cell extracts (27, 49). HeLa cells infected with a lentivirus carrying scrambled or RACK1-targeting shRNA were expanded up to 80% confluence. Cells were then trypsinized and lysed for 45 min at 4°C in 10 mM HEPES (pH 7.6), 10 mM K acetate, 0.5 mM Mg acetate, 5 mM DTT, and protease inhibitor (Promega). Lysates were homogenized by passing through a 27-gauge, 3/4-in syringe needle and were clarified by centrifugation at 18,000 × *g* for 1 min. Protein concentrations were determined by bicinchoninic acid (BCA) quantification assays (Thermo Fisher Scientific). Lysates not immediately used for the assay were aliquoted and stored at −80°C. To make translation fully dependent on exogenously added mRNA, lysates were treated with 15 U/ml S7 micrococcal nuclease (Roche) and 0.75 mM CaCl_2_ and were incubated at 22°C for 7 min. EGTA (2 mM) was added to terminate the reaction.

For each sample in the translation assay, 6 μl of S10 extract was mixed with 1.2 μl of master mix (125 mM HEPES, 10 mM ATP, 2 mM GTP, 200 mM creatine phosphate, 0.2 mM amino acid mixture without methionine [Promega], 0.25 mM spermidine, 20 mM l-methionine, 50 mM K acetate, 2.5 mM Mg acetate, 20 U of RNasin [Promega], and 0.5 μg of purified reporter-encoding mRNA). The mixture for the *in vitro* translation reaction was then incubated for 90 min at 30°C. The Dual-Glo luciferase assay kit (Promega) was used to read firefly and Renilla luciferase outputs with a GloMax luminometer (Promega). For the rescue experiments, 100 ng of purified maltose-binding protein (MBP)-RACK1 (wild type or mutant) and 100 ng of purified MBP per assay were added to the lysates and were incubated for 5 min at room temperature before the assay was performed.

### Mutagenesis on RACK1-encoding plasmids.

We performed mutagenesis on RACK1-encoding plasmids by PCR using the QuikChange II site-directed mutagenesis kit (Agilent Technologies). We purchased primer oligonucleotides carrying the mutations from Sigma-Aldrich. We set the PCR program at 30 s at 95°C, followed by 18 cycles of 30 s at 95°C, 1 min at 55°C, and 15 min at 68°C.

### MBP-RACK1 purification.

We transformed chemically competent E. coli BL21(DL3) to produce MBP-RACK1 proteins. To induce the expression of the recombinant proteins, we supplemented the medium with 0.5 mM isopropyl-β-d-thiogalactopyranoside (IPTG) for 3 h. We then lysed the bacteria by sonication in a column buffer composed of 20 mM Tris-Cl (pH 7.4), 200 mM NaCl, 1 mM EDTA, 1 mM Na azide, and 1 mM DTT.

After clarification, we loaded the lysates onto Poly-Prep chromatography columns (Bio-Rad) containing 1 ml of amylose resin (New England BioLabs) preequilibrated with column buffer. After several washes with column buffer, we proceeded to protein elution with 10 mM maltose, and fractions of 300 μl were collected. We diluted the fractions enriched in the recombinant proteins in a buffer made of 20 mM Tris-Cl, 2 mM CaCl_2_, and 100 mM NaCl at pH 8.8 and incubated them overnight on a rotating wheel with 1 μg of Factor Xa Protease (New England BioLabs) per 50 μg of MBP-RACK1 in order to separate RACK1 from the MBP tag.

We then dialyzed the proteins against the buffer required for the subsequent *in vitro* translation assay (10 mM HEPES [pH 7.6], 10 mM K acetate, 0.5 mM Mg acetate, 5 mM DTT). After SDS-PAGE and Coomassie blue staining, ImageJ, v1.48, was used to determine the concentrations of the purified proteins. The recombinant proteins were stored at +4°C for future use.

### *In vitro* ribosome interaction assay (*i*RIA).

Ribosome interaction experiments were performed as described previously (32). Briefly, 96-well plates (Nunc) were coated overnight at 4°C with 50 μg/well of RACK1-downregulated HeLa cell lysate diluted in 50 μl of PBS. The coating solution was removed, and nonspecific sites were blocked for 1 h at room temperature with 5% BSA in PBS. Plates were washed with 200 μl/well PBS–0.05% Tween 20. Previously purified recombinant proteins (MBP, wild-type RACK1-MBP, or R36D K38E mutant RACK1-MBP) were biotinylated by using the EZ-Link Micro Sulfo-NHS-LC biotinylation kit (Thermo Scientific) according to the manufacturer's instructions. The biotinylated recombinant proteins were added at 0.5 μg per well in 50 μl PBS. The recombinant proteins were incubated on the coated total cell extract for 2 h at room temperature. After washing with PBS–0.05% Tween 20, HRP-conjugated streptavidin in PBS–0.05% Tween 20 was added to wells for 30 min at room temperature in a final volume of 50 μl. After washes, 3,3′,5,5′-tetramethylbenzidine (TMB) was used according to the manufacturer's protocol (Sigma-Aldrich) to detect streptavidin peroxidase activity. HCl at 1 N was used as the stop solution. The absorbance at 450 nm was read on an Infinite F200 multiwell plate reader (Tecan).

### Copurification of His-eIF4E interactors.

For each sample, we incubated 8 μg of recombinant His-tagged eIF4E (Cayman Chemical) with 120 μg of HeLa cell lysates (in 10 mM HEPES [pH 7.6], 10 mM K acetate, 0.5 mM Mg acetate, 5 mM DTT, and protease inhibitor from Promega). We performed the experiments under conditions of endogenous mRNA digestion by micrococcal nuclease. We compared control samples with samples reconstituted with exogenous pRL-TK mRNA with an *in vitro*-synthesized poly(A) tail under *in vitro* translation conditions. After incubation of the recombinant eIF4E on the lysates, we loaded the samples onto Talon metal affinity resin (Clontech Laboratories) that had been equilibrated with PBS. After 30 min on a rotating wheel at +4°C, we removed the supernatant and washed the resin in 10 mM HEPES (pH 7.6), 10 mM K acetate, and 0.5 mM Mg acetate. Proteins were eluted by boiling the samples in Laemmli buffer plus 20 mM EDTA. We detected purified proteins by SDS-PAGE and Western blotting.

### Copurification of MBP-RACK1 interactors.

We first induced the expression of MBP-RACK1 constructs in E. coli BL21(DL3) as described above. We then proceeded to purify the proteins through MBP pulldown. We incubated total-HEK293-cell lysates on the resin binding the constructs. We used 1.2 mg of lysate per 30 μg of the purified proteins. After three washes in column buffer (20 mM Tris-Cl [pH 7.4], 200 mM NaCl, 1 mM EDTA, 1 mM Na azide, 1 mM DTT), we eluted the MBP recombinant proteins in column buffer plus 10 mM maltose. We concentrated the proteins by trichloroacetic acid (TCA) precipitation and resuspended the resulting pellets in Laemmli buffer. We performed SDS-PAGE on the samples, followed by Coomassie blue staining and Western blotting for the proteins of interest.

### Ribosome profiles.

We performed ribosome profiling as described previously (50) with some optimizations on HEK293 cells. Briefly, 48 h after transfection with RACK1-HaloTag constructs, we replated the cells. After 4 h, we pretreated the cells for 20 min with 0.36 mM cycloheximide (Sigma-Aldrich). Then we washed the cells with PBS and proceeded with lysis in 50 mM Tris-Cl (pH 7.5), 100 mM NaCl, 30 mM MgCl_2_, 0.1% Igepal CA-630, 0.36 mM cycloheximide, 40 U/ml RNasin, and protease inhibitor cocktail. After clarification, we determined the RNA concentrations in the samples by reading the absorbance at 260 nm with a BioPhotometer Plus instrument (Eppendorf).

We loaded the equivalent of 10 optical density (OD) units for each sample on 15-to-30% sucrose gradients in 50 mM Tris-acetate (pH 7.5), 50 mM NH_4_Cl, 12 mM MgCl_2_, and 1 mM DTT, and we centrifuged the samples at 4°C in an SW41 Ti swinging-bucket rotor (Beckman Coulter) for 3 h 30 min at 39,000 rpm. We recorded the absorbance at 254 nm by use of BioLogic LP software (Bio-Rad), and 40S, 60S, and 80S peaks were defined. We concentrated the collected fractions (1 ml per fraction) by TCA precipitation. We washed pellets with TCA wash buffer (70% acetone, 20% ethanol, 50 mM Tris-Cl [pH 8.8]), dried them, and resuspended them in sample buffer for SDS-PAGE and subsequent Western blotting.

### RACK1-HaloTag purification.

We transfected HEK293 cells with RACK1-HaloTag or control green fluorescent protein (GFP) constructs. After 48 h from transfection, we lysed the cells in 150 mM NaCl, 50 mM Tris-HCl, 4 mM MgCl_2_, 1% Triton X-100, 0.05% Igepal CA-630, and protease inhibitor cocktail. After clarification and determination of protein concentrations with BCA, we loaded 1 mg of each sample onto equilibrated HaloLink resin (Promega) according to the manufacturer's instructions. When specifically testing the relevance of the integrity of mRNA for RACK1 protein interactions, we either added 40 U/ml RNasin to the lysates or treated the samples with 15 U/ml S7 micrococcal nuclease and 0.75 mM CaCl_2_ at 22°C for 7 min. We then inhibited micrococcal nuclease activity by adding 2 mM EGTA to the lysates.

We incubated the lysates on the resin for 90 min on a rotating wheel at room temperature. We then washed the resin three times with 150 mM NaCl, 50 mM Tris-HCl, 4 mM MgCl_2_, and 0.05% Igepal CA-630 and incubated again for 90 min with 20 U of AcTEV protease (Invitrogen) in 200 μl of tobacco etch virus (TEV) buffer on a rotating wheel at room temperature. We loaded samples and performed SDS-PAGE, followed by Western blotting for RACK1 and copurifying proteins. In parallel, liquid chromatography (LC)–electrospray ionization (ESI)–tandem mass spectrometry (MS-MS) analysis was performed to confirm RACK1 interactions with ribosomal proteins.

### Time-lapse microscopy.

After 24 h from transfection with wild-type or R36D K38E mutant RACK1-HaloTag, HEK293 cells were incubated with the HaloTag TMR ligand (Promega) for 30 min at 37°C. Then the medium was changed, and the cells were incubated in complete DMEM for 15 min to remove excess fluorescent dye. After the medium was changed again, the fluorescence in the cells was monitored for the subsequent 24 h with a Nikon Eclipse Ti microscope (Nikon Instruments), and images were taken every hour.

### Cell cycle analysis.

HEK293 cells expressing RACK1-targeting shRNA or RACK1-HaloTag constructs were analyzed under conditions of active growth by comparison with controls. Before the analysis, we supplemented the medium with 10 μM bromodeoxyuridine (BrdU) (from the APC BrdU flow kit, produced by BD Biosciences) for 6 h. After that, if the cells were transfected with RACK1-HaloTag, they were incubated with the HaloTag TMR ligand (Promega) for 30 min at 37°C according to the manufacturer's instructions. Then we changed the medium to eliminate any excess of HaloTag TMR dye. We trypsinized and centrifuged the cells for 5 min at 500 × *g*. After that, we utilized the reagents and protocol from the APC BrdU flow kit to resuspend, fix, and permeabilize cells, to degrade the DNA in order to expose BrdU epitopes, to mark BrdU by staining with an allophycocyanin (APC)-conjugated antibody, and to stain total DNA with 7-aminoactinomycin D (7-AAD).

We used a FACSAria III system (BD Biosciences) to measure fluorescence in cells and FlowJo, v10.3 (Tree Star), to perform analysis.

### Single-cell puromycin incorporation analysis.

To measure protein synthesis in single living cells, we adapted a protocol to detect the incorporation of puromycin by fluorescent staining (51). Forty-eight hours post-transient transfection of wild-type or mutant RACK1-HaloTag in HEK293 cells, we replated the cells. We treated the cells with either the p38 mitogen-activated protein kinase (MAPK) inhibitor SB203580 (Sigma-Aldrich) at 10 μM or dimethyl sulfoxide (DMSO) for 8 h. Then, 30 min prior to fixation, we added the HaloTag TMR ligand (Promega) to the medium, followed by supplementation with 9.2 μM puromycin (Gibco) 20 min prior to fixation. We changed the medium 10 min prior to fixation in order to remove any excess HaloTag TMR dye. We incubated fixed samples with an antipuromycin antibody (clone 12D10; Millipore) for 1 h at room temperature and then with an Alexa Fluor 488-labeled goat anti-mouse secondary antibody (Invitrogen) according to the manufacturer's instructions.

We quantified fluorescence in the cells with a FACSAria III system (BD Biosciences) and selected and analyzed populations with FlowJo, v10.3 (Tree Star).
